# *Burkholderiaceae* and Multidrug Resistance Genes Are Key Players in Resistome Development in a Germfree Soil Model

**DOI:** 10.1128/mSystems.00988-21

**Published:** 2021-11-02

**Authors:** Yuping Cao, Yigal Achmon, Sima Yaron, Bupe A. Siame, Ka Yin Leung

**Affiliations:** a Biotechnology and Food Engineering, Guangdong Technion—Israel Institute of Technology, Shantou, China; b Faculty of Biotechnology and Food Engineering, Technion—Israel Institute of Technology, Haifa, Israel; c Department of Biology, Trinity Western University, Langley, British Columbia, Canada; University of Illinois at Chicago

**Keywords:** resistome, germfree soil, multidrug resistance genes, microbiome, metagenomics

## Abstract

Assembly of a resistome in parallel with the establishment of a microbial community is not well understood. Germfree models can reveal microbiota interactions and shed light on bacterial colonization and resistance development under antibiotic pressure. In this study, we exposed germfree soil (GS), GS with diluted nontreated soil (DS), and nontreated soil (NS) to various concentrations of tetracycline (TET) in a nongermfree environment for 10 weeks, followed by 2 weeks of exposure to water. High-throughput sequencing was used to profile bacterial communities and antibiotic resistance genes (ARGs) in the soils. The initial bacterial loads were found to shape the profiles of bacterial communities and the resistomes. GS and DS treated with TET and the same soils left untreated had similar profiles, whereas NS showed different profiles. Soils with the same initial bacterial loads had their profiles shifted by TET treatment. Multidrug resistance (MDR) genes were the most abundant ARG types in all soils, with multidrug efflux pump genes being the discriminatory ARGs in GS regardless of different TET treatments and in GS, DS, and NS after TET. Furthermore, MDR genes were significantly enriched by TET treatment. In contrast, tetracycline resistance genes were either absent or low in relative abundance. The family *Burkholderiaceae* was predominant in all soils (except in NS treated with water) and was positively selected for by TET treatment. Most importantly, *Burkholderiaceae* were the primary carrier of ARGs, including MDR genes.

**IMPORTANCE** This is the first study to examine how resistomes develop and evolve using GS. GS can be used to study the colonization and establishment of bacterial communities under antibiotic selection. Surprisingly, MDR genes were the main ARGs detected in GS, and TET treatments did not positively select for specific tetracycline resistance genes. Additionally, *Burkholderiaceae* were the key bacterial hosts for MDR genes in the current GS model under the conditions investigated. These results show that the family *Burkholderiaceae* underpins the development of resistome and serves as a source of ARGs. The ease of establishment of *Burkholderiaceae* and MDR genes in soils has serious implications for human health, since these bacteria are versatile and ubiquitous in the environment.

## INTRODUCTION

Microorganisms in the environment produce many antibiotics, harbor thousands of antibiotic resistance genes (ARGs), and are continually evolving as part of an ongoing evolutionary arms race. Researchers have isolated and investigated many antibiotic producers in an effort to discover new antibiotics with potential clinical applications. Moreover, studies on the function, distribution, regulation, and evolution of ARGs can be used to develop strategies aimed at combating and reversing the emergence of multiple-antibiotic-resistant microorganisms. Early studies, which were generally based on axenic cultures, unveiled resistance phenotypes and genotypes in various human pathogens (1–3). Furthermore, these studies revealed the genetic background of ARGs and the mechanisms mediating their dissemination, such as in the cases of *bla*_NDM-1_, *optrA*, and *mcr-1* resistance genes (4–6). However, the methodologies were inadequate to interrogate ARGs from a broader ecological perspective, i.e., the resistome. The resistome, a term first proposed by D'Costa et al. in 2006 (7), is the collection of all the ARGs from pathogenic and nonpathogenic microorganisms in genomes or mobile genetic elements (MGEs) of a defined microbial community. More specifically, it includes intrinsic ARGs that are detected in all taxonomically related taxa, acquired ARGs that are present natively in other taxonomically unrelated taxa, proto-ARGs that are nonfunctional and distinct from clinically significant ARGs, and silent ARGs that are nonfunctional and can be identified by comparison based on homologous sequences (8).

Recently, researchers have used next-generation sequencing (NGS), which bypasses the limitation of unculturable bacteria, to study resistomes. ARG databases, such as SARG, ARDB, MEGARES, and CARD, have been constructed and widely used (9–12). Together with molecular biology techniques, NGS has also evolved into functional metagenomics, to characterize and validate novel ARGs in the absence of prior knowledge of targeted sequences (13, 14). Numerous studies have revealed the ubiquity of resistomes, and the similarity or difference in resistomes recovered from different types of samples, e.g., a resistome shared between soil and humans, was reported (15). Feng and colleagues (16) reported that in the human gut, genes conferring resistance to tetracycline, macrolide-lincosamide-streptogramin (MLS), and multiple drugs are the most abundant ARG types. Similarly, MLS, multidrug, and tetracycline resistance genes are the most prevalent ARG types in black soil, which is important for crop production in China (17). Also, a high level of tetracycline, multidrug, and erythromycin ARGs are shared by swine, poultry, and human feces (18). The tight connections among humans, animals, and environment urge us to study the resistome from the One Health perspective (19). Investigation into each compartment can reflect its contribution to the overall evolution of the resistome.

Researchers have used samples from different sources, such as manure, sewage sludge, and reclaimed water, to gradually elucidate the forces that shape resistomes in different environments (20–22). Simulation of resistome evolution is the next frontier, since this is the key to predicting and reversing the spread of antibiotic resistance. Lab modeling can examine factors that are influential in resistome evolution, such as the introduction of antimicrobials, heavy metals, aromatics, biochar, and plants (23–27). Xiong et al. (28) used a chicken model to show that therapeutic doses of chlortetracycline reduced the amount of Escherichia species, the main carrier of multidrug resistance (MDR) genes, in the gut of chickens. Lu and Lu (27) also found that Azolla imbricata plants could effectively reduce the number and diversity of ARGs in soils by the elimination of antibiotics and heavy metals. The resistome is further shaped by the interplay among members in the microbiome. Duan and colleagues (29) found that inoculation of Bacillus subtilis into composting manure decreased the relative abundance of ARGs, MGEs, and pathogenic bacteria. Overall, the evolution of the resistome results from a diversity of biotic and abiotic factors, either external or internal, that affect the composition of bacterial community and/or resistome.

Previous studies profiled the bacterial communities and resistomes and/or correlated various factors to the changes in these profiles. Despite promising results, not many studies have looked at initial bacterial colonization and new resistome formation in niche areas with no prior detectable microorganisms. Additionally, the effect of environmental selection on those bacterial communities and resistomes remains a mystery. A germfree model provides a unique lens through which to explore the establishment of a resistome. By exposing a germfree model to a defined environment, one can capture the assembly of a new microbiome and a new resistome with and without exposure to stresses that might serve as a force for shaping both the microbiome and the resistome. For several decades, germfree animals have been used to study the interaction between host and its microbiota, especially the gut flora that is closely associated with metabolic, inflammatory, and neuropsychiatric diseases (30). Recently, germfree animals have been introduced to microbiome and resistome studies. Thomas et al. (31) reported the prevalent phyla in 18-day-old germfree chickens that were exposed to cecal contents, e.g., *Bacteroidetes*, *Firmicutes*, and *Actinobacteria*. Using a germfree piglet model, Liu and Wang (32) found that the transplantation of the fecal microbiota resulted in higher prevalence and abundance of ARGs and the occurrence of ARGs that were originally undetected. Of note, tetracycline, multidrug, and MLS were the most dominant ARG types in the germfree piglets that received microbiota transplantation (32). Thus, germfree models can help unravel how resistomes become established and evolve, which will also aid prediction of resistome changes and the development of reverse strategies.

Currently, resistome studies based on a germfree soil (GS) model are lacking. There are differences in the abundance and composition of bacterial communities found in soils and guts. The evolution of a soil resistome is shaped by numerous factors that include the presence of various pollutants, antibiotic producers, and different antibiotic concentrations. Additionally, the effects of antibiotics, such as tetracycline (TET), on newly established and mature bacterial communities are unknown. This study aimed to answer two fundamental questions: (i) which key bacteria, ARGs, and ARG hosts appear first during the formation of a new resistome in soils? and (ii) how is the resistome affected by different initial bacterial loads and by exposure to different antibiotic concentrations? In order to address these questions, we developed and validated a GS model to mimic conditions existing before bacteria assemble in soils and then followed the development of resistome in the presence of TET. GS with no detectable bacteria or bacterial DNA was exposed to the surrounding environment to allow colonization by exogenous bacteria and to support the establishment of a new resistome. Moreover, GS mixed with diluted nontreated soil (DS) and nontreated soil (NS) gave insights into the resistome development from the interplay between indigenous and exogenous bacteria. Subsequently, the soils were challenged with various concentrations of TET to allow selection of TET-resistant bacteria and ARGs and reveal the diverse evolutionary events of the soil resistomes. We expected that intrinsic ARGs and their bacterial hosts would be the key to resistome development in the GS, since intrinsic ARGs are generally ancient, omnipresent, and multifunctional (33). We also postulated that TET would positively select the tetracycline resistance genes and their hosts in soils. To account for potentially significant differences (e.g., abundance and composition) between communities and resistomes of NS and GS, we metagenomically profiled the bacterial communities, resistomes, and bacterial hosts of ARGs during different stages of resistome development. Our results suggest that *Burkholderiaceae* carrying MDR genes and multidrug efflux pumps play key roles in resistome establishment and in resistome development under TET pressure in the soils.

## RESULTS

### Establishment of bacterial communities using 16S rRNA gene sequencing.

**FIG 1:**
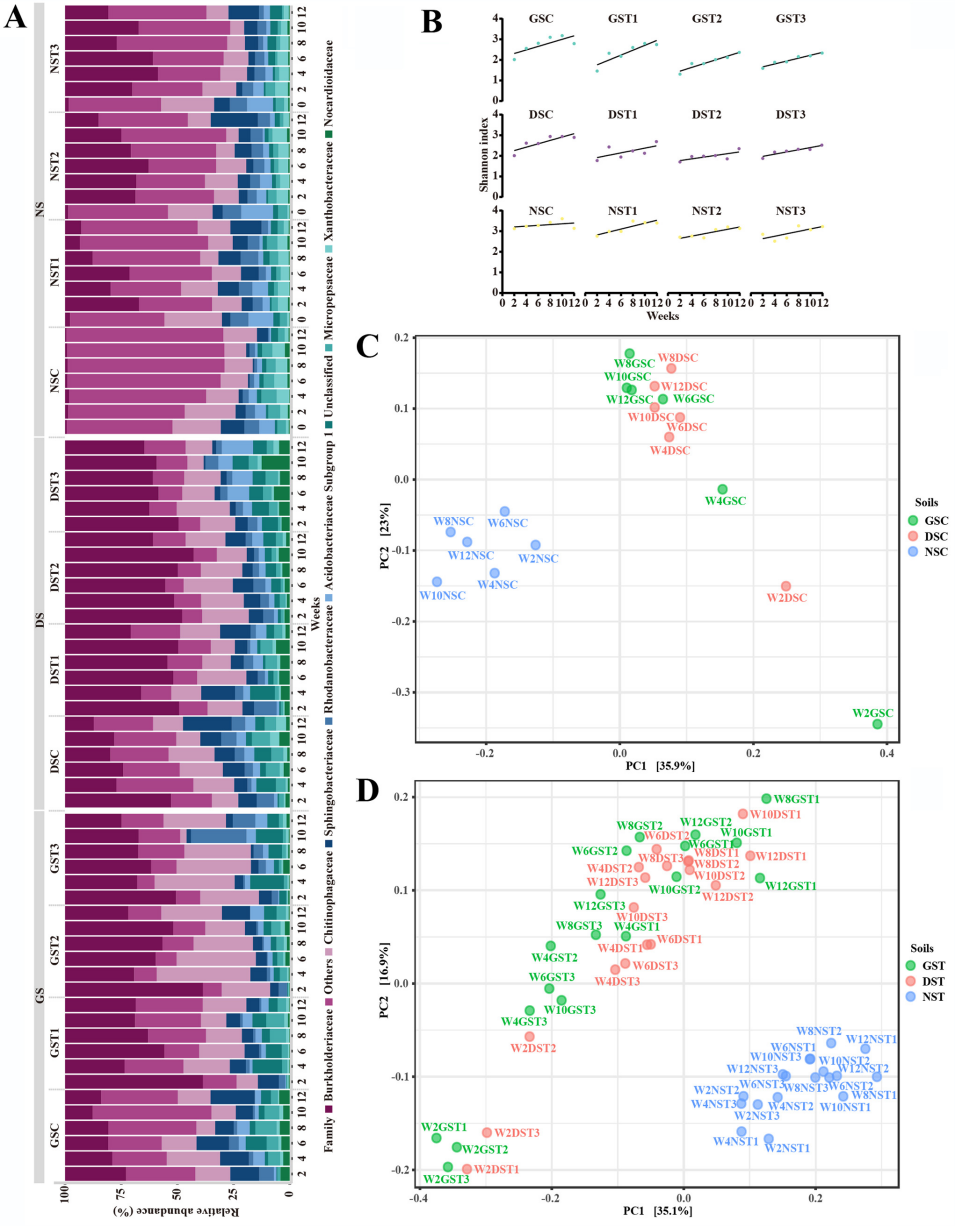
Bacterial communities in a germfree soil model revealed by 16S rRNA analysis over a 12-week period. In this model, germfree soil (GS), GS with diluted nontreated soil (DS), and nontreated soil (NS) were irrigated with TET (4 μg/ml) for 2 weeks, followed by decreasing (T1), unchanging (T2), or increasing (T3) TET concentration every 2 weeks for 8 weeks. After 10-weeks of exposure to TET, all soils were irrigated with sterile water for the next 2 weeks (Table 1). Details of the bacterial communities in control groups (GSC, DSC, and NSC) and TET treatment groups (GST1 to -T3, DST1 to -T3, and NST1 to -T3) are given in Data Set S1, tab 2. GST, DST, and NST denote combined results from T1, T2, and T3, respectively. Very little or no DNA was found in GSC, DSC, GST1 to -T3, and DST1 to -T3 at the start of the experiment (week 0), and hence, no bacterial compositions are presented for GST, DST, or NST. Statistical analyses were performed using the Friedman test and multiple Wilcoxon matched-pairs signed-rank test, with the Benjamini-Hochberg method to control the FDR (*q* < 0.1). (A) Eight predominant families were identified, and the relative abundances are shown, with *Burkholderiaceae*, *Chitinophagaceae*, and *Sphingobacteriaceae* as the most abundant. (B) Alpha diversity analysis (Shannon index) showing bacterial diversities increased over the 12-week study period across all soils. Regression lines were generated using Shannon index. (C and D) Beta diversity analysis showed that GS and DS results clustered together but were removed from those for NS. Data were visualized by principal-coordinate analysis (PCoA) of the bacterial families from soil samples with TET treatments. Unweighted UniFrac, as a distance metric, and ANOSIM, as a statistical test, were used for the analysis (*P < *0.05). Labels of individual soil samples are given (e.g., W2GST1 denotes results from week 2 of GST1). (C) Control groups (*P < *0.05); (D) TET treatment groups (*P < *0.05).

16S rRNA gene sequencing revealed the colonization and development of bacterial communities in soils. In GS, where no bacteria were initially present, bacterial communities originated from external bacteria, whereas bacterial communities in DS and NS developed from indigenous bacteria and/or exogenous bacteria. Samples were named according to the time points of collection, soil types, and treatments. For example, W2GSC refers to GS that were collected after a 2-week (W2) exposure to water (C) (Table 1). Similarly, GST refers to all the GS exposed to TET, including samples exposed to decreasing (T1), unchanging (T2), and increasing concentrations (T3) of TET. Negligible amounts of DNA, insufficient for the sequencing process, were found in GSC, GST1 to GST3, DSC, and DST1 to DST3 at the start of the experiment (week 0). Thus, only the bacterial communities of NSC and NSC (T1 to T3) at week 0 were included for comparison (Fig. 1A). In all the soils, the eight most abundant taxa identified at the bacterial family level were *Burkholderiaceae*, *Chitinophagaceae*, *Sphingobacteriaceae*, *Rhodanobacteraceae*, *Acidobacteriaceae* subgroup 1, *Micropepsaceae*, *Xanthobacteraceae*, and *Nocardioidaceae* (Fig. 1A). These families accounted for 27 to 91% of the sequences. A ninth group remained unclassified (0.03 to 15%) according to the SILVA database. Other taxa with low abundances were merged and defined as “others,” with details described in Data Set S1, tab 2. Overall, soils with different initial bacterial loads and TET treatments differed in bacterial community profiles (Fig. 1A). Comparative analysis of the relative abundance of the above taxa revealed that GS was similar to DS and both were different from NS, which had the largest number of significantly different taxa (multiple Wilcoxon matched-pairs signed-rank test; *q *< 0.1) (Data Set S1, tab 3). The abundance of *Burkholderiaceae*, *Xanthobacteraceae*, and *Nocardioidaceae* showed significant differences among GS, DS, and NS with the same treatments, with either water only or TET (Friedman test, *P < *0.05) (Fig. S1A and B). Of note, the relative abundance of *Burkholderiaceae* in GS and DS was significantly higher than in NS, regardless of the treatment groups (multiple Wilcoxon matched-pairs signed-rank test; *q *< 0.1) (Fig. S1A and B; Data Set S1, tab 3). Also, TET treatments positively selected *Burkholderiaceae* in the three soils, i.e., GS, DS, and NS (Fig. S1C to E; Data Set S1, tab 3). DESeq2, LEfSe, and edgeR analyses showed similar results. In addition, *Burkholderiaceae* had the highest detection threshold in the core microbiome analyses of all soil types (Fig. S1F). When TET treatments were stopped after week 10, established bacterial communities were only slightly affected or reshaped afterwards. This lack of change in bacterial communities after discontinuing the TET selection pressure may result from the stability of developed resistomes (Fig. 1A). Therefore, *Burkholderiaceae* were key to the colonization of soils by bacterial communities and were positively selected for under TET treatments.

**TABLE 1:** Experimental design of soil with different bacterial loads undergoing various TET treatments

Sample type^*a*^	TET concn (μg/ml) at wks:					
	0–2	2–4	4–6	6–8	8–10	10–12
C	0	0	0	0	0	0
T1	4	2	1	0.5	0.25	0
T2	4	4	4	4	4	0
T3	4	8	16	32	64	0

a Control groups and treatment groups were designed for soils of different initial bacterial loads: germfree soil (GS), GS with diluted nontreated soil (DS), and nontreated soil (NS). Control groups (GSC, DSC, and NSC) were irrigated with autoclaved deionized water without TET for 12 weeks. TET treatment groups (GST, DST, and NST) were irrigated with decreasing (T1), unchanging (T2), or increasing (T3) concentrations of TET for 10 weeks. Autoclaved water without TET was used to irrigate all soil samples in weeks 11 and 12.

10.1128/mSystems.00988-21.1 FIG S1 (A to E) The top eight taxa were significantly different among different soils (Friedman test; *P < *0.05). Taxa with lowercase letters (“a”, “b”, and “c”) retained strong differentiation after FDR correction (multiple Wilcoxon matched-pairs signed-rank test; *q *< 0.1). (A) Three control groups; (B) 3 treatment groups. Letters indicate the pairwise comparisons of soils with the same treatment but with different initial bacterial loads; e.g., “a” and “b” indicate GSC versus DSC and GSC versus NSC, respectively. (C) Four GS samples; (D) 4 DS samples; (E) 4 NS samples. “a”, “b,” and “c” indicate the comparison of control (C) versus treatment (T1, T2, and T3) samples, respectively; e.g., “b” refers to C versus T2. (F) *Burkholderiaceae* had the highest detection threshold in the core microbiome analyses of all the soil samples without NS samples from week 0. Bacterial families that remained unchanged in composition across all groups were analyzed, with 20% as the sample prevalence and 0.01% as the relative abundance in MicrobiomeAnalyst. (G to K) Shannon indexes that were significantly different between soils in pairwise tests are labeled with asterisks (Friedman test; *q *< 0.1). (G) Three control groups; (H) 3 treatment groups; (I) 4 GS samples; (J) 4 DS samples; (K) 4 NS samples. (L to N) Beta diversity analysis, visualized by PCoA, to show the changes of bacterial communities in the soils with the same initial bacterial loads but different TET treatments, e.g., GSC, GST1, GST2, and GST3. The data were visualized by PCoA, with unweighted UniFrac as a distance metric and ANOSIM (*P < *0.05) as a statistical test. (L) 4 GS samples (*P < *0.05); (M) 4 DS samples (*P < *0.05); (N) 4 NS samples (*P < *0.05). Download FIG S1, TIF file, 2.3 MB. Copyright © 2021 Cao et al. 2021 Cao et al. https://creativecommons.org/licenses/by/4.0/ This content is distributed under the terms of the Creative Commons Attribution 4.0 International license.

10.1128/mSystems.00988-21.4 DATA SET S1 (Tab 1) Information of 16S rRNA sequencing after DADA2. (Tab 2) Changes of bacterial composition (family level) based on relative abundance. (Tab 3) Statistical tests on the relative abundance of taxa and Shannon index. (Tab 4) Relative abundance of ARG types over time. (Tab 5) Relative abundance of ARG subtypes over time. (Tab 6) Statistical tests on the relative abundance of ARG types and relative abundance of total ARGs. (Tab 7) Pairwise statistical tests on the resistomes profiles. (Tab 8) Details of assembly, contigs, and ORFs. (Tab 9) ARG subtypes and the corresponding hosts. (Tab 10) ARG types and the corresponding hosts. (Tab 11) Variations of predominant hosts carrying MDR genes over time. Download Data Set S1, XLSX file, 0.5 MB. Copyright © 2021 Cao et al. 2021 Cao et al. https://creativecommons.org/licenses/by/4.0/ This content is distributed under the terms of the Creative Commons Attribution 4.0 International license.

To study the diversity of bacterial communities and the differences in bacterial composition among the soils, alpha diversity analysis and beta diversity analysis were performed at the bacterial family level. In the treatment of control soils, Shannon indexes were similar in GSC and DSC but were significantly lower than that of NSC (Friedman test; *q *< 0.1) (Fig. S1G). In TET-treated soils, GST had a Shannon index similar to that of DST, which was significantly lower than that of NST (Friedman test; *q *< 0.1) (Fig. S1H). TET treatments significantly reduced bacterial diversity in all the soils relative to the corresponding control soils treated only with water except GST1 and NST1, which were treated with decreasing TET (Friedman test; *q *< 0.1) (Fig. S1I to K). In general, unchanged TET was the most influential treatment, resulting in the lowest Shannon indexes in soils (Fig. S1I to K). Examining soil samples individually shows that the bacterial diversity tended to increase with time (Fig. 1B). Within the three soils, GS generally had the lowest bacterial diversity whereas NS had the highest diversity at week 2. After week 8, the Shannon indexes were relatively stable in GSC and DSC and were similar to that of NSC at week 2, suggesting that they all possessed bacterial communities with a similar diversity. Therefore, we concluded that 10 weeks was sufficient time to study the colonization and development of soil microbiomes. Discontinuation of TET treatments after week 10 generally led to an increase in bacterial diversity (Fig. 1B).

For beta diversity, principal-coordinate analysis (PCoA) based on all the taxa unveiled significantly different bacterial communities among soils with different initial bacterial loads and treatments (*P < *0.05; analysis of similarity [ANOSIM]) (Fig. 1C and D). In controls treated with water (GSC and DSC), bacterial composition differed from that of NSC (Fig. 1C) (*P* < 0.05). Similar results were observed in TET-treated groups, where NST communities were distant from those in GST and DST (Fig. 1D) (*P < *0.05). Additionally, clear separations of the bacterial communities were observed among soils with the same initial bacterial loads but with different TET treatments (Fig. S1L to N) (*P < *0.05). The results suggested that initial bacterial loads and TET treatments accounted for the dissimilarities in soil bacterial communities.

### Metagenomic sequencing to demonstrate the development of resistomes.

**FIG 2:**
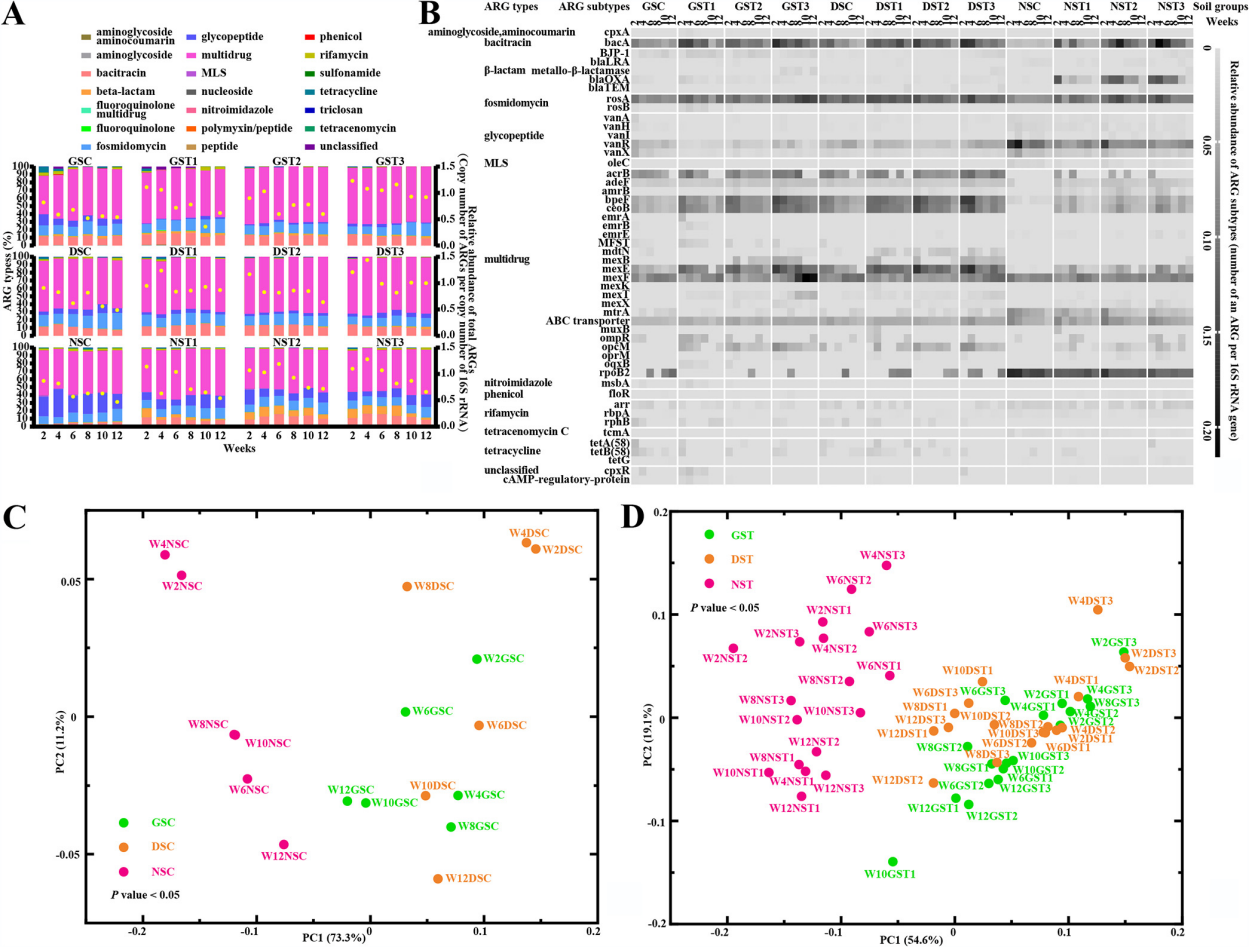
Metagenomic analysis of ARGs in different soils over the 12-week study period. In this model, germfree soil (GS), GS with diluted nontreated soil (DS), and nontreated soil (NS) were irrigated with TET (4 μg/ml) for 2 weeks, followed by decreasing (T1), unchanging (T2), or increasing (T3) TET concentration every 2 weeks for 8 weeks. After 10 weeks of exposure to TET, all soils were irrigated with sterile water for the next 2 weeks (Table 1). Details of ARG types and subtypes in the control groups (GSC, DSC, and NSC) and the TET treatment groups (GST1 to -T3, DST1 to -T3, and NST1 to -T3) are described further in Data Set S1, tab 4 and tab 5, respectively. GST, DST, and NST denote combined results from T1, T2, and T3. (A) ARG types and the relative abundance of total ARGs varied in different soils. Total ARGs decreased over time (right *y* axis), and the relative abundance of ARG types (left *y* axis) showed a predominance of the multidrug resistance genes. (B) Heat map of variations of ARG subtypes based on the relative abundance of ARG subtypes in the control groups (GSC, DSC, and NSC) and TET treatment groups (GST1 to -T3, DST1 to -T3, and NST1 to -T3). The top 50 ARG subtypes in all the soils over the examined period are shown. Multidrug resistance (MDR) gene types and multidrug efflux pumps, e.g., *mexF*, were favored in the germfree soil model. Details of all ARG subtypes are described in Data Set S1, tab 5. (C and D) Beta diversity analysis (*P < *0.05) on soil resistomes in 3 control groups (C) and 3 treatment groups (D). These results showed that GS and DS clustered together but were away from NS. Data were visualized by PCoA on the relative abundance of ARG subtypes, with Bray-Curtis as a distance metric and PERMANOVA as a statistical test (*P < *0.05). Labels indicate individual soil samples; e.g., W2GST1 denotes results for GST1 from week 2.

Metagenomic sequencing confirmed the relative abundance of ARGs and ARG hosts, which were used to profile the development of resistomes. The relative abundance of an ARG subtype was calculated as the copy number of ARG subtype per copy number of 16S rRNA gene. Calculation of the total abundance of ARGs, i.e., the sum of all the relative abundances of ARG subtypes (Fig. 2A), revealed that ARGs decreased steadily in soils treated with water over the 12-week period. The value ranged between 0.52 and 0.82 in GSC, 0.49 and 0.91 in DSC, and 0.47 and 0.87 in NSC. Similarly, soils treated with TET showed a gradual decrease in overall abundance of ARGs during the 10-week exposure to TET. Upon discontinuation of TET at the end of week 10, the overall abundance of ARGs rebounded only in GST1, indicating that the established resistomes were generally stable at the community level. Furthermore, no significant difference was found among soils receiving the same treatment, i.e., TET or water, despite having different initial bacterial loads (Friedman test, *q *< 0.1) (Fig. S2A and B). On the other hand, TET consistently raised the overall abundance of ARGs significantly, regardless of initial bacterial loads (Fig. S2C to E).

10.1128/mSystems.00988-21.2 FIG S2 (A to E) Relative abundance of total ARGs that were significantly different between soils in pairwise tests were labeled with asterisks (Friedman test, *q *< 0.1). (A) Three control groups; (B) 3 treatment groups; (C) 4 GS samples; (D) 4 DS samples; (E) 4 NS samples. Results showed that increased TET raised the relative abundance of total ARGs significantly, relative to the control soils treated with water. (F to J) The ARG types were significantly different among different soils (Friedman test, *P < *0.05). ARG types with small letters (“a,” “b,” and “c”) retain strong differentiation after FDR correction (multiple Wilcoxon matched-pairs signed-rank test; *q *< 0.1). (F) Three control groups; (G) 3 treatment groups. Letters indicate the pairwise comparisons of soils with the same treatment but different initial bacterial loads; e.g., “a” and “b” indicate GSC versus DSC and GSC versus NSC, respectively. (H) Four GS samples; (I) 4 DS samples; (J) 4 NS samples. “a,” “b,” and “c” indicate the comparison of control (C) versus treatment (T1, T2, and T3) samples, respectively; e.g., “b” refers to C versus T2. (K to M) Beta diversity analysis showed that TET treatments resulted in significant differences in resistome profiles in soils with the same initial bacterial load. Data were visualized by PCoA on the relative abundance of ARG subtypes, with Bray-Curtis as a distance metric and PERMANOVA as a statistical test (*P < *0.05). (K) Four GS samples (*P < *0.05); (L) 4 DS samples (*P < *0.05); (M) 4 NS samples (*P < *0.05). Labels indicate individual soil samples; e.g., W2GST1 indicates results GST1 from week 2. Download FIG S2, TIF file, 1.3 MB. Copyright © 2021 Cao et al. 2021 Cao et al. https://creativecommons.org/licenses/by/4.0/ This content is distributed under the terms of the Creative Commons Attribution 4.0 International license.

In total, 98 ARG subtypes were characterized, representing 21 ARG types: multidrug, bacitracin, fosmidomycin, rifamycin, β-lactam, glycopeptide, phenicol, tetracycline, nitroimidazole, MLS, fluoroquinolone, fluoroquinolone/multidrug, peptide, aminoglycoside, aminoglycoside/aminocoumarin, tetracenomycin C, triclosan, nucleoside, sulfonamide, polymyxin, and unclassified (Data Set S1, tabs 4 and 5). Furthermore, compositional changes in ARG types were revealed and the relative abundance of ARG types were compared (Fig. S2F to J). In general, multidrug, glycopeptide, fosmidomycin, and bacitracin were the predominant ARG types in all the soils (Fig. 2A). NST had a significantly higher level of β-lactam relative to GST and DST in TET treatments (Fig. S2G). Comparative analysis based on ARG types revealed that GS was similar to DS, both of which were different from NS that had the largest number of significantly different ARG types (multiple Wilcoxon matched-pairs signed rank test, *q *< 0.1) (Data Set S1, tab 6). Noticeably, multidrug was the most abundant ARG type in all the soils during the investigated period. Compared to the soils treated only with water, TET-treated soils had a significantly higher relative abundance of MDR genes, such as in GST3, DST3, NST2, and NST3 (Fig. S2H to J). Surprisingly, there were no significant differences in tetracycline resistance genes in soils after TET exposure.

### ARG subtype analysis.

Resistome profiles were reconstructed based on the relative abundance of ARG subtypes (Fig. 2B). Surprisingly, ARG subtypes from MDR genes, especially multidrug efflux pumps, ABC transporters, and *mexF*, dominated in all the soils and remained high in relative abundance over the study period. Interestingly, *mexF*, which can confer tetracycline resistance, underwent positive selection under increased TET treatment. For example, the relative abundance of *mexF* increased with the TET concentration in GST3 over the 10-week exposure. Despite discontinuation of TET treatment after week 10, the abundance of *mexF* remained high until the end of week 12, unlike other ARG subtypes, such as *bacA*, *bpeF*, and *ceoB*. In contrast, the relative abundance of *tetA*(58) and *tetB*(58) decreased with time or remained low in the soils (Fig. 2B). Furthermore, we identified other multidrug efflux pumps as discriminatory ARGs that gave unique occurrence patterns of resistomes in the soils (Table 2). The discriminatory ARGs detected in GSC but not in NSC encoded multidrug (*acrB*, *ceoB*, *emrB*, and major facilitator superfamily transporter [MFST]), tetracycline [*tetA*(58)], rifamycin (*rphB*), phenicol (*clbB* and *floR*), fosmidomycin (*rosB*), unclassified (cyclic AMP [cAMP]-regulatory protein), aminoglycoside [*aac*(6′)-31], and sulfonamide (*sul2*) resistance (Table 2). Of note, high relative abundances of the efflux pump gene *ceoB* (mean ± SD, 0.03684 ± 0.01071) and *acrB* (0.05351 ± 0.02808) were observed in GSC. The discriminatory ARGs found in GST but not in NST were those for multidrug (*mdtN* and MFST) and rifamycin (*rphB*) resistance (Table 2), all of which were in relatively low abundance. Compared to the corresponding soils with water, discriminatory ARGs that existed exclusively in GS, DS, and NS after TET treatments were mainly MDR genes (Table 2). Taken together, these results suggest that MDR genes (especially multidrug efflux pumps) and not tetracycline resistance genes play a fundamental role in the resistome establishment even in the presence of TET in soils.

**TABLE 2:** Discriminatory ARGs that exhibited unique occurrence patterns of resistomes in soils^*a*^

Pairwise comparison	First comparator		Second comparator	
	Resistance type	Gene or protein	Resistance type	Gene or protein
GSC vs. NSC	Aminoglycoside	*aac*(6′)-31	Beta-lactam	*bla*_LRA_
	Fosmidomycin	*rosB*	Glycopeptide	*vanI*
	Multidrug	MFST	MLS	*oleC*
	Multidrug	*ceoB*	Multidrug	*emrE*
	Multidrug	*acrB*	Multidrug	*efpA*
	Multidrug	*emrB*	Tetracenomycin C	*tcmA*
	Phenicol	*clbB*		
	Phenicol	*floR*		
	Rifamycin	*rphB*		
	Sulfonamide	*sul2*		
	Tetracycline	*tet*A(58)		
	Unclassified	cAMP-regulatory protein		

GST vs. NST	Multidrug	MFST	Glycopeptide	*vanI*
	Multidrug	*mdtN*	MLS	*oleC*
	Rifamycin	*rphB*	Tetracenomycin C	*tcmA*
			Tetracycline	*tetG*

GSC vs. GST1	Aminoglycoside	*aac*(6′)-31	Aminoglycoside	*ksgA*
	Glycopeptide	*vanA*	Aminoglycoside, Aminocoumarin	*cpxA*
	Multidrug	*mdtN*	Beta-lactam	*bla*_LRA_
	Multidrug	*rpoB2*	Beta-lactam	*penA*
	Phenicol	*clbB*	Fluoroquinolone	*emrR*
	Rifamycin	*rbpA*	Fluoroquinolone, multidrug	*mdtK*
	Sulfonamide	*sul2*	Multidrug	*mexT*
	Tetracycline	*tetG*	Multidrug	*mdtA*
			Multidrug	*opcM*
			Multidrug	*mdtH*
			Multidrug	*baeR*
			Multidrug	*tolC*
			Multidrug	*oqxB*
			Unclassified	H-NS

GSC vs. GST2	Multidrug	*mdtN*	Multidrug	*opcM*
	Phenicol	*clbB*	Multidrug	*mexT*
	Sulfonamide	*sul2*	Multidrug	*mexB*
			Multidrug	*mexX*

GSC vs. GST3	Phenicol	*clbB*	Beta-lactam	Metallo-β-lactamase
	Sulfonamide	*sul2*	Multidrug	*amrB*
			Multidrug	*opcM*
			Multidrug	*mexT*
			Multidrug	*oprM*
			Multidrug	*mexX*
			Multidrug	*mexB*

DSC vs. DST1	Multidrug	MFST	Aminoglycoside, aminocoumarin	*cpxA*
			Beta-lactam	*bla*_LRA_
			Beta-lactam	*bla*_TEM_
			Fluoroquinolone, multidrug	*mdtK*
			Fosmidomycin	*rosB*
			Multidrug	*emrE*
			Multidrug	*mdtH*
			Multidrug	*muxB*
			Multidrug	*mexN*
			Multidrug	*mvaT*
			Multidrug	*mexK*
			Multidrug	*muxC*
			Multidrug	*mexD*
			Multidrug	*emrD*
			Multidrug	*mexB*
			Multidrug	*mipA*
			Multidrug	*mexA*
			Multidrug	*oprM*
			Multidrug	*marA*
			Multidrug	*mexW*
			Multidrug	*oprJ*
			Peptide, polymyxin	*arnA*
			Phenicol	*clbB*
			Triclosan	*opmH*
			Unclassified	cAMP-regulatory protein

DSC vs. DST2	Multidrug	*ompR*	Beta-lactam	*bla*_TEM_
	Multidrug	MFST	Glycopeptide	*vanS*
	Rifamycin	*arr*	Multidrug	*amrB*
			Multidrug	*mexX*
			Multidrug	*mexB*
			Multidrug	*oprM*

DSC vs. DST3	Multidrug	MFST	Beta-lactam	Metallo-β-lactamase
	Tetracycline	*tet*A(58)	Beta-lactam	*bla*_TEM_
			Multidrug	*amrB*
			Multidrug	*mexB*
			Multidrug	*oprM*
			Multidrug	*mexX*

NSC vs. NST1			Multidrug	*amrB*
			Multidrug	*ceoB*
			Multidrug	*opcM*
			Multidrug	*mexX*
			Multidrug	*acrB*

NSC vs. NST2			Multidrug	*amrB*
			Multidrug	*ceoB*
			Multidrug	*opcM*
			Multidrug	*mexX*
			Phenicol	*floR*

NSC vs. NST3			Beta-lactam	Metallo-β-lactamase
			Multidrug	*amrB*
			Multidrug	*ceoB*
			Multidrug	*mexB*
			Multidrug	*oprM*
			Multidrug	*opcM*
			Multidrug	*mexX*
			Multidrug	*mexT*
			Phenicol	*floR*
			Tetracycline	*tet*A(58)

a ExtraARG revealed the discriminatory ARGs that were found exclusively in each soil in the pairwise comparison. MFST, major facilitator superfamily transporter. The multidrug efflux pumps were a key determinant of soil resistome profiles in GSC and GST, and in GS, DS, and NS after TET.

In addition, beta diversity analysis with principal-component analysis (PCA) and PERMANOVA (*P < *0.05) was performed to study the dissimilarities in resistomes (Fig. 2C and D; Fig. S2K to M). As expected, GSC and DSC were significantly different from NSC (Fig. 2C; Data Set S1, tab 7). When TET was applied, there were significant differences between the soils treated with TET but having different initial bacterial loads, i.e., GST versus DST, GST versus NST, and DST versus NST (Fig. 2D; Data Set S1, tab 7). The patterns revealed in the above results were similar to those revealed by 16S rRNA sequencing (Fig. 1C and D). Under the same bacterial load, significant differences were observed between the soils with water and TET treatments, e.g., GSC versus GST1, GST2, and GST3 (Fig. S2K to M; Data Set S1, tab 7). Overall, we see that soil resistomes are shaped by initial bacterial loads and TET treatments.

### ARGs and their hosts.

**FIG 3:**
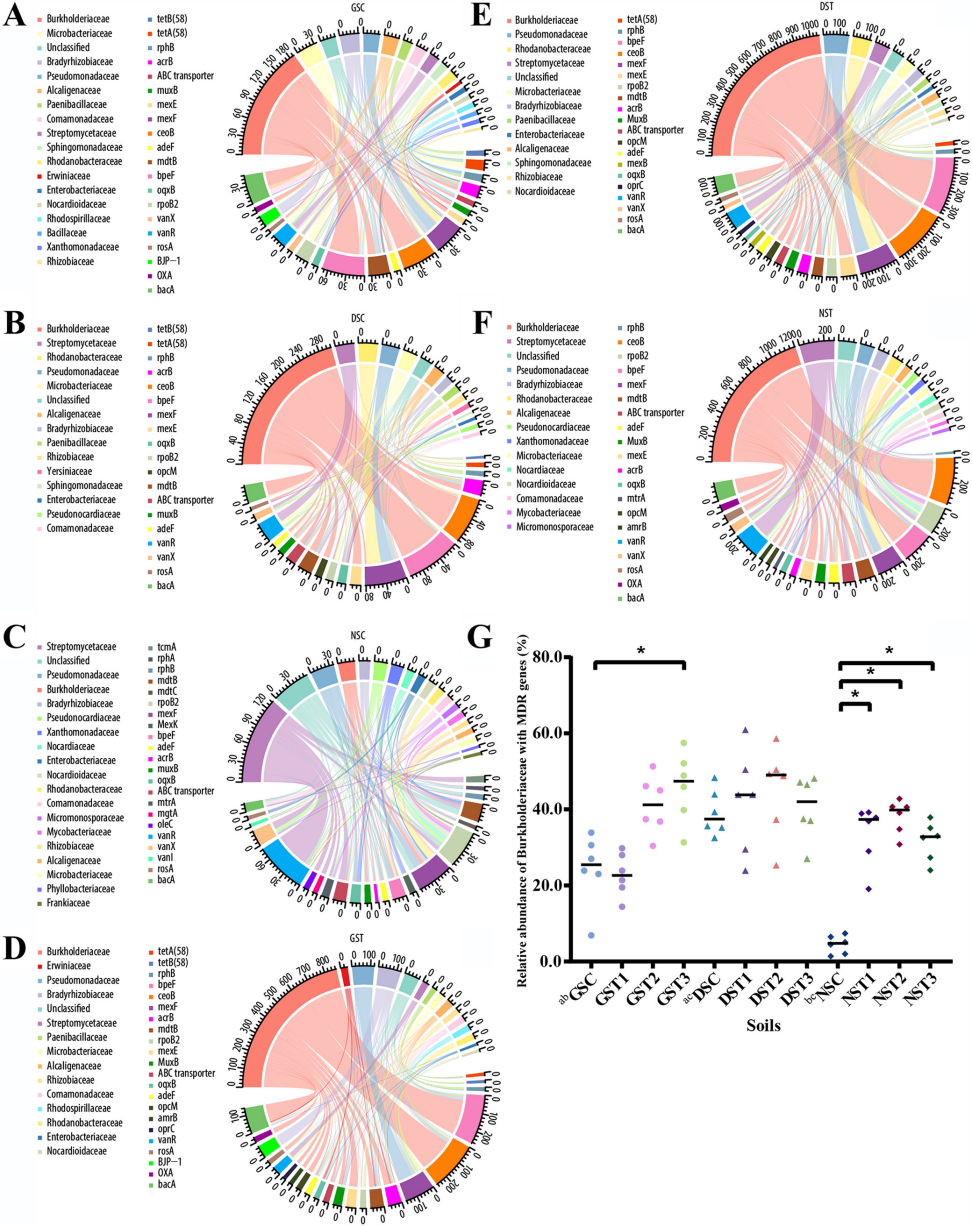
ARGs and their bacterial hosts in germfree soil (GSC and GST1 to -T3), GS with diluted nontreated soil (DSC and DST1 to -T3), and nontreated soil (NSC and NST1 to -T3). Host bacterial families and ARGs with relative abundances of ≥1% are shown. The outermost circle represents the actual counts of each host and ARG subtype. The relationship between ARGs and the corresponding hosts is indicated by lines in the inner circle. Details of the profiles are listed in Data Set S1, tabs 9, 10, and 11. *Burkholderiaceae* were the main carriers of ARGs and MDR genes. *Burkholderiaceae* with MDR genes were also positively selected by TET treatments. (A to F) ARG host profiles in the different soils. (A) GSC; (B) DSC; (C) NSC; (D) GST; (E) DST; (F) NST. (G) Relative abundances of *Burkholderiaceae* with MDR genes that were significantly different between soils in pairwise analyses (Friedman test; *q *< 0.1). Asterisks shows significant differences, and “a,” “b,” and “c” indicate comparisons between GSC and DSC, GSC and NSC, and DSC and NSC, respectively.

Taxonomic classification of the bacteria hosting the ARGs is crucial to our understanding of the origin and development of resistomes; thus, the contigs carrying ARG open reading frames (ORFs) were used to classify the bacterial hosts. Clean sequences were assembled into 56,282,585 contigs with an average length of 1,040 bp and an average *N*_50_ of 1,590 bp. A total of 94,453,457 ORFs were obtained, from which 9,362 ARG ORFs and 9,095 contigs carrying ARG ORFs were found with lengths ranging from 200 bp to 901,224 bp, a median length of 575 bp, and an average length of 6,280 bp (Fig. S3), and 9,243 bacterial taxa were identified (Data Set S1, tabs 8 and 9). Subsequently, a major part of the ARG host profile was represented by the observed ARG subtypes (relative abundance ≥ 1%), and their bacterial hosts at the family level (relative abundance ≥ 1%) (Fig. 3A to F). The complete ARG host profiles are described in Data Set S1, tabs 9 and 10. In GSC and DSC, the most predominant ARG host was the family *Burkholderiaceae*, making up 31.97% and 45.09% of the profiles, respectively (Fig. 3A and B; Data Set S1, tab 9). In comparison, *Streptomycetaceae* (26.15%) was the most prevalent host in NSC, although the family *Burkholderiaceae* was also represented as one of the most abundant hosts (5.30%) (Fig. 3C; Data Set S1, tab 9). In the presence of TET, *Burkholderiaceae* continued to dominate the ARG host profiles in soils; 46.22% in GST, 48.67% in DST, and 42.36% in NST (Fig. 3D to F; Data Set S1, tab 9). Other major hosts in the TET treated soils were *Pseudomonadaceae*, *Bradyrhizobiaceae*, *Streptomycetaceae*, *Microbacteriaceae*, *Alcaligenaceae*, *Rhodanobacteraceae*, and *Nocardioidaceae*, regardless of the initial bacterial loads (Fig. 3D to F).

10.1128/mSystems.00988-21.3 FIG S3 Distribution of contigs carrying ARGs. The contig lengths range from 200 bp to 901,224 bp, with a median length of 575 bp and an average length of 6,280 bp. Download FIG S3, TIF file, 0.2 MB. Copyright © 2021 Cao et al. 2021 Cao et al. https://creativecommons.org/licenses/by/4.0/ This content is distributed under the terms of the Creative Commons Attribution 4.0 International license.

We found that the family *Burkholderiaceae* carried diverse ARG subtypes and was the main carrier of MDR genes. In the soils treated with water, *Burkholderiaceae* with MDR genes represented the largest proportion in GSC (25.45%) and DSC (39.28%), while *Streptomycetaceae* carrying glycopeptide ARGs were the most predominant in NSC (14.66%) (Data Set S1, tab 10). Similarly, *Burkholderiaceae* carrying MDR genes were the most abundant among TET-treated soils, i.e., 37.10% in GST, 42.62% in DST, and 34.53% in NST (Data Set S1, tab 10). Examining the abundance over time revealed that *Burkholderiaceae* harboring MDR genes were the most prevalent in all the soils during the experimental period, e.g., 24.22% ± 9.42% in GSC and 39.13% ± 5.98% in DSC. The only exception was NSC, which had an average relative abundance of 4.48% ± 2.39% (Data Set S1, tab 11). Furthermore, the abundance of *Burkholderiaceae* with MDR genes in GSC and DSC was significantly higher than that of NSC and was positively selected for by TET in GS and NS but not in DS (Friedman test, *q *< 0.1) (Fig. 3G), correlated with the results of 16S rRNA analysis (Fig. S1A and C to E). As indicated by 16S rRNA analysis, *Burkholderiaceae* had the highest detection threshold in the core microbiome analyses of all soils (Fig. S1F).

In order to decipher differences in resistome evolution between newly developed and mature bacterial communities, we compared the relative abundance of the major hosts of MDR genes in GST3 and NST3, soils treated with increasing TET (Data Set S1, tab 11). As expected, *Burkholderiaceae* carrying MDR genes dominated the ARG-host profiles during the study period and ranged from 31.33% to 57.53% and 24.00% to 37.86% in GST3 and NST3, respectively. In addition to *Burkholderiaceae*, we identified other hosts of MDR genes specific to the sample type; e.g., *Alcaligenaceae* and *Enterobacteriaceae* were found exclusively in GST3, and *Streptomycetaceae* and *Nocardioidaceae* were unique to NST3.

The gene *mexF*, capable of conferring tetracycline resistance, was abundant at the start of the experiment but was further selected for in GST3 (Fig. 2B). It reached a high abundance by week 8 and was maintained over weeks 10 and 12, with values of 0.163, 0.216, and 0.200, respectively. This was the most abundant ARG subtype at these sampling time points (Fig. 2B; Data Set S1, tab 5). Noticeably, some of the observed hosts of *mexF* were the primary taxa as revealed by 16S rRNA sequencing, i.e., *Rhodanobacteraceae* at week 2 (2.85%), *Rhizobiaceae* at week 4 (2.22%), *Burkholderiaceae* at week 6 (38.27%), *Rhodanobacteraceae* (10.02%), and *Rhizobiaceae* (1.45%) at week 12 (Data Set S1, tab 2). However, none of the observed hosts of *mexF* in GST3 at week 8 (*Rhodospirillaceae* and *Pseudomonadaceae*) and week 10 (*Rhodospirillaceae*, *Pseudomonadaceae*, and *Alcaligenaceae*) were detected by 16S rRNA analysis (Data Set S1, tab 2). The results suggest that vertical gene transfer was the determinant in resistome development.

In summary, *Burkholderiaceae*, which were prevalent in the bacterial communities and carried MDR genes, underpinned resistome development under TET selection in soils. TET treatment also shaped the resistomes by positively selecting *Burkholderiaceae* with MDR genes.

## DISCUSSION

Many studies have looked at already-established resistomes from diverse sources or tried to correlate intrinsic and environmental factors with resistome evolution in order to understand the spread of ARG and to combat antibiotic resistance. However, the process by which the resistome itself develops has remained less studied. Microorganisms exploit the environment to colonize and outcompete others using ARGs (34); thus, resistomes can develop during and following bacterial colonization (Fig. 1A and 2B). Of note, soil resistomes are mainly determined by bacterial community structures (35). Inevitably, commonly used soil models with mature bacterial communities fail to unveil the assembly of a new resistome. To fill this gap, we constructed soil models with different initial bacterial loads, mimicking the bacterial populations that initiate the development of resistomes with or without TET selection. Combining NGS and bioinformatics, we profiled changes in the bacterial communities, resistomes, and bacterial hosts of ARGs during different stages of resistome development in soils. This analysis demonstrated the importance of MDR genes, especially multidrug efflux pumps, and their major host, *Burkholderiaceae*, in resistome development even in the presence of TET in soils.

The composition of bacterial communities in soils was affected by the initial bacterial loads and TET treatments. More specifically, bacterial isolation and 16S rRNA gene amplification revealed that the bacterial load in GS before exposure to the environment and TET was extremely low. The main source of bacteria in GS could be external and possibly from the environment (aerosols and human intervention). In DS, the communities might have developed by the interplay of indigenous and exogenous bacteria. Last, NS with established bacterial communities was used for comparison. With water treatment, *Burkholderiaceae* prevailed as the most common bacteria in GSC and DSC. These results are consistent with other studies that found *Burkholderia* to be ubiquitous and to occupy diverse ecological niches, such as air, soil, water, and humans (36–38). Compared to NSC, GSC and DSC had significantly enriched amounts of *Burkholderiaceae*, implying a vital role of *Burkholderiaceae* in the initial colonization of bacterial communities and development of resistomes. The predominant phyla in DSC were *Actinobacteria*, *Acidobacteria*, *Bacteroidetes*, *Cyanobacteria*, *Gemmatimonadetes*, *Planctomycetes*, *Proteobacteria*, and *Verrucomicrobia*. Similarly, Yan et al. (39) found that *Proteobacteria*, *Bacteroidetes*, and *Verrucomicrobia* predominated after incubation of GS created by gamma irradiation with normal soils. Our alpha diversity analysis showed that DSC, with a low bacterial load, needed 8 weeks to develop into maturity (Fig. 1B), in agreement with the results of Yan and colleagues (39). The introduction of TET reduced Shannon indexes in soils (Fig. S1I to K), which was in line with the findings that TET reduced bacterial diversity in normal soil and that the reduction was TET concentration dependent (40). It is not surprising that NS that had mature communities remained stable and had fewer dissimilarities in bacterial composition than GS and DS. Similarly, adults have mature gut microbiotas that are believed to be stable even with antibiotic administration (41). In addition, TET positively selected for *Burkholderiaceae*, suggesting that *Burkholderiaceae* contribute to resistome development in soils. The result reflects those of Podnecky et al., who found that *Burkholderia* has multidrug efflux pumps conferring resistance to tetracycline (42).

After quantifying ARGs and identifying discriminatory ARGs, we observed that MDR genes, especially multidrug efflux pumps, were the core of resistome in GS. In addition, the presence of other ARG types at week 2 indicated that MDR genes were not the only environmental option for resistome. More specifically, in GSC, ARGs encoding multidrug efflux pumps were prevalent in resistome profiles during the investigated period, e.g., ABC transporter, *acrB*, *adeF*, *ceoB*, *bpeF*, and *mexF*. Moreover, several pumps, i.e., *ceoB*, MFST, *acrB*, and *emrB*, resulted in the unique occurrence pattern in the GSC resistomes (Fig. 2B; Table 2). The results might be explained by the fact that multidrug efflux pumps are ancient and ubiquitous and can have diverse biological functions (33, 43). For example, *acrB* was detected in *Burkholderia* spp. recovered from uriniferous soils (44). Also, *mexF* has been identified in air, soil, marine, and humans (45–48). In addition, *mexF* was detected exclusively across the 50-year succession age of the retreating glacier where microbes were the initial colonizers, and an increased trend of relative abundance in soil resistomes was reported (49). The pumps tend to have a wide range of substrates in addition to antibiotics; they can extrude toxic compounds such as metabolites and heavy metals, and signaling molecules to facilitate cell-to-cell communication (43). They are believed to underlie the origin of antibiotic resistance (33). It has been found that *mexF* involved the extrusion of toxic kynurenine which is an intermediate in the degradation of tryptophan and a precursor of anthranilate in Pseudomonas aeruginosa (50). Additionally, *mexF* has been linked to the transport of a precursor of quinolone signaling in Pseudomonas (51). In other studies, *acrB* facilitated the arrest of growth after Escherichia coli intercellular contact, which is characterized by the secretion of tRNA nuclease CdiA (52, 53). Another function of *acrB* is in conferring resistance to bile salts, which enables *Enterobacteriaceae* to survive the environment of the mammalian gut (54). Moreover, MFST (*rmrAB*) was used by Rhizobium etli to withstand flavonoids of plants (55). Therefore, it is possible that MDR genes (especially multidrug efflux pumps), probably due to their ubiquity and multifunctionality, are foundational to resistome development in soils. Interestingly, pumps believed to be immobilized in the chromosome, have been increasingly associated with MGEs, such as the plasmid-mediated RND (resistance-nodulation-division) pump that was recovered from Klebsiella pneumoniae (56). The normalization of ARGs with 16S rRNA genes might affect the exact quantification of ARGs, since multiple copies of 16S rRNA genes are commonly present in bacteria. Future studies can consider a single copy gene such as *gyrA* or *recA* for the normalization of ARG amount.

By evaluating the effects of initial bacterial loads and TET treatments on soil resistomes, we found that bacterial communities resulting from diverse initial bacterial loads structured the resistomes in soils (Fig. 1C and D and 2C and D). This is consistent with the finding that bacterial community compositions determine the occurrence of soil resistomes across habitats (35). Moreover, we observed that TET shaped the resistomes by positively selecting the MDR genes. Similarly, Xiong et al. (28) showed that TET shaped the resistomes in broiler guts by changing specific ARG subtypes. More importantly, we observed that resistome development in soils under TET was driven by MDR genes but not necessarily by ARGs that are commonly relevant to clinical environments, such as tetracycline resistance genes (Fig. S2F to J). The relative abundance of MDR genes was generally high, but the abundance of tetracycline resistance genes remained low during TET treatments (Fig. 2B). This contrasts with the results reported by Xiong et al. (28), in which high doses of chlortetracycline decreased the abundance of MDR genes by reducing the main carrier, Escherichia, but at the same time increased the abundance of tetracycline resistance genes in broiler guts. One explanation for the discrepancy may be the positive selection on bacteria carrying MDR genes by TET, or the outperformance of tetracycline resistance genes in broiler guts after long-term use of TET in the broiler industry (28). It can also be explained by the different microbial composition in the gut and in the soil, since *Enterobacteriaceae* were rare in the investigated soil samples. Taken together, the results call for a further interrogation into the effects of MDR genes from the environment, as they have a critical role in resistome development under TET selection in soils. In addition to MDR genes, TET treatments enriched GS, DS, and NS with the bacitracin ARGs, whereas beta-lactam ARGs were enriched in NS (Fig. S2H to J). Interestingly, *mexT*, the regulator of the *mexE-mexF-oprN* multidrug efflux system, was enriched only in soils with increasing TET concentrations (GST3 and DST3). Additionally, *mexE* was enriched in the early stages of TET exposure, whereas *mexF* was predominant much later in GST3 and DST3. Therefore, the soil resistomes appears to be influenced by both the initial bacterial community and the duration of antibiotic exposure.

Based on the taxonomic classification of bacterial hosts of ARGs, we observed consistent profiles of bacterial communities and resistomes (Fig. 1A, 2B, and 3A to F). Regardless of the treatments, the ARG host profiles of GS were like DS, but different from NS profiles. It is possible that the ARG hosts in GS came from NS, such as *Streptomycetaceae* carrying *rpoB2*; however, the possibility that the hosts originated from the environment cannot be excluded. *Burkholderiaceae* carrying MDR genes were the basis for resistome development (GSC) under the influence of TET in soils. Further, *Burkholderiaceae* was the primary carrier of the most predominant ARG type, MDR genes, and was also capable of harboring diverse ARG subtypes; *Burkholderiaceae* with MDR genes were positively selected for by TET (Fig. 3A to G). These results explain the differences in resistomes between our soils and broilers in the presence of TET (28). Of note, the result that *Burkholderiaceae* carry pumps like that encoded by *bpeF* corresponds to the previous finding that *Burkholderia*, the type genus of the family *Burkholderiaceae*, encodes BpeEF-OprC, which uses TET as a major substrate (42, 57). Interestingly, *Burkholderiaceae* were also shown to be the vital hosts for ARGs in activated sludge from wastewater treatment plants (58).

*Burkholderiaceae* are common soil inhabitants, and this family contains pathogens of plants, humans, and animals. Its genus *Burkholderia* is of clinical concern; it causes glanders, melioidosis, and pulmonary infections that lead to high mortality rates in cystic fibrosis patients (59). There are approximately 165,000 cases of melioidosis caused by environmental *Burkholderia* in the world per year (60). Currie and colleagues (36) identified aerosolized *Burkholderia* under stormy weather conditions and provided clear circumstantial evidence for the inhalation of *Burkholderia* by the patient. In our study, the *Burkholderiaceae* blooms in GS, DS, and NS after TET treatments were most likely from the environment, since some ARGs carried by *Burkholderiaceae* were not present in GSC, DSC, and NSC (e.g., *amrB* and *mexX*). Indeed, further studies are needed, since sequence depth in the present study might not be enough to unveil all the genes in the soils. Additionally, *Burkholderiaceae* carrying bacitracin-type ARG, which were absent in NSC, were present in NS after TET treatments, suggesting that the introduction of a single antibiotic enabled the communities to be resistant to diverse antibiotics. The secondary modulation of soil resistomes could be due to TET that turned on the antibiotic production in communities.

Generally, *Burkholderia* infections are difficult to treat due to the bacteria’s resistance to frontline beta-lactams and to polymyxins, treatments of last resort in humans (57). The importance of *Burkholderiaceae* should not be underestimated, because this family has a significant presence in soil resistomes, as revealed in the current study, wide distribution, broad-host-range pathogenesis, and notorious antibiotic resistance. It is imperative that further investigation of its role in soil resistomes be conducted, along with the evaluation of its potential contributions to the shared resistome between soils and humans (15). Also, risk assessments on antibiotic residues, especially TET, in clinically relevant environments are required to prevent selection for *Burkholderiaceae*. The effects of various factors, such as climate, TET residues, soil texture, and ARG dynamics (such as single nucleotide polymorphisms that cannot be identified by DeepARG), should be investigated further.

When examined individually, GST3 with increasing TET revealed that *mexF*, a pump from MDR genes that can confer TET resistance (61), was initially abundant and further selected for by TET and remained in large amounts after TET was stopped (Fig. 2B). Similarly, the absolute abundance of *mexF* significantly increased in soils that were exposed to organic manure, chlortetracycline, and ciprofloxacin (62). Noticeably, several bacterial hosts of *mexF*, at weeks 2, 4, 6, and 12, were the dominant taxa at the same time point, as revealed by 16S rRNA sequencing (Data Set S1, tab 2), which suggests positive selection for resistant bacteria. Meanwhile, there are inconsistencies in the results at week 8 and 10 between 16S rRNA and metagenomic sequencing which may result from the horizontal transfer of *mexF*. Interestingly, Norberg et al. (47) reported a mobile *mexF* in an IncP plasmid recovered from marine microbial biofilms. Maintenance of the high level of *mexF* after stopping TET treatments might be due to the fact that *mexF* did not impair fitness of the host during competition (50). These results reaffirmed the vital role of multidrug efflux pumps in the development of soil resistomes challenged with antibiotics. The overexpression of pumps can confer antibiotic resistance to clinically relevant bacteria (63), which is advantageous to the host bacteria since it imparts multiantibiotic resistance with reduced fitness costs via a single pump (64). It is also possible that MGE-associated pumps “prime” the soil to facilitate resistome development. Future studies can use inhibitors of multidrug efflux pumps to explore the reasons for the enrichment of nonspecific ARGs relative to antibiotic specific ARGs. Moreover, future studies in germfree models should explore the effect of other antibiotics (apart from tetracycline) on soil resistome development. It will be interesting to apply culturomics to examine the resistance phenotypes of bacterial communities with enriched multidrug efflux pumps.

## MATERIALS AND METHODS

### Construction of soil microcosms and TET treatments.

Commercial loam soil (Nord Agri, Latvia), suitable for the growth of most plants, with a pH range of 5.0 to 6.5 and containing 50 to 260 mg/liter nitrogen, 50 to 260 mg/liter phosphate, and 50 to 340 mg/liter potassium, was directly used (NS). This NS was then used to construct soil microcosms of different initial bacterial loads, i.e., GS and DS. To obtain GS, NS was sterilized three times at 121°C for 2 h in autoclave bags (Alex Red, Israel). To confirm the absence of live bacteria, soils were plated out on Luria broth agar (LBA); no colonies appeared after incubating GS on LBA plates at 37°C for 7 days. To validate the absence of bacterial DNA in GS, bacterial DNA was extracted using a NucleoSpin soil kit (Macherey-Nagel, Germany) and amplified by PCR with primers 515F and 907R (V4 and V5 regions) (65, 66). DS was prepared as described by Yan et al. (39) with minor modifications. Specifically, 10 ml of NS and 90 ml of autoclaved deionized water were mixed and serially diluted in sterile deionized water to reach a final concentration of 10^−4^ (vol/vol). Then, 15 ml of the final diluted soil suspension was thoroughly mixed with 300 g of GS to generate DS. Therefore, we obtained soil with various bacterial loads, i.e., no bacterial load in GS, relatively low bacterial load in DS, and normal bacterial load in NS.

Different soils (300 g) with different bacterial loads were transferred to plastic potting containers (with drainage holes). The pots were placed in an outdoor shelter protected from rain to allow bacterial colonization and establishment of resistomes. Four replicates of each soil sample were prepared. In control groups without antibiotic treatments, three types of soil samples (GSC, DSC, and NSC) were irrigated daily with 100 ml sterile (autoclaved) deionized water for 12 weeks. In TET treatment groups, soil samples were irrigated daily with 100 ml of sterile water containing TET (T3383; Sigma‐Aldrich, USA). The TET starting concentration was 4 μg/ml, because it is the TET resistance breakpoint for most of the pathogens (Clinical and Laboratory Standards Institute [CLSI] [83]). Different concentrations of TET were introduced to examine the development of microbiome and resistome under different levels of ecological pressure. TET treatments lasted for 10 weeks. During the TET treatments, the TET concentrations were decreased (T1) in GST1, DST1, and NST1 soils and increased (T3) in GST3, DST3, and NST3 soils, every 2 weeks (Table 1). The TET concentration remained unchanged (T2) in GST2, DST2, and NST2 soils for 10 weeks (Table 1). After 10 weeks, all soil samples were irrigated daily with 100 ml autoclaved deionized water for another 2 weeks to assess the fitness costs of the established resistomes.

### Soil sampling and DNA extraction.

For sampling, 2 g of soil was randomly sampled at a depth of 2 cm in each container every 2 weeks, from week 2 to week 12. A total of 8 g of soil was collected from each sample consisting of four replicates. Soil was collected with a sterile spoon, pooled, and stored in sterile 50-ml tubes (Eppendorf, Germany) at −20°C. The individual soil samples were mixed thoroughly and separately prior to bacterial DNA extraction using a NucleoSpin soil kit (Macherey-Nagel, Germany). If necessary, multiple extractions were performed to obtain at least 1 μg bacterial DNA from each sample. Quantification of DNA was carried out using a Qubit double-stranded-DNA (dsDNA) broad-range (BR) assay kit (Invitrogen, USA).

### 16S rRNA profile.

Genes encoding the V4-V5 regions of 16S rRNA were amplified using primers 515F (5′-GTGCCAGCMGCCGCGG-3′) and 907R (5′-CCGTCAATTCMTTTRAGT-3′) followed by sequencing on an Illumina 2500 platform (BGI, China), which generated paired-end 300-bp sequences. The primers included an Illumina adapter, pad, and linker sequences. The PCR was carried out as follows: 94°C for 3 min, 30 cycles of 94°C for 30 s, 53°C for 45 s, and 72°C for 45 s, and then 72°C for 10 min. Quality control was performed on 16S rRNA sequences as follows. (i) A 25-bp sliding window was used to determine the quality of the sequences; if the average quality of a sequence fell below 20 bp, the sequence was trimmed. The remaining sequence was used only if the length was more than 75% of its original length. (ii) Sequences with contamination of adapters, ambiguous bases (N), and low complexity (10 identical consecutive bases) were also removed. After quality control, additional sequencing was performed if there were fewer than 30,000 sequences in each sample.

The clean sequences were analyzed using QIIME2 and MicrobiomeAnalyst (67, 68). The primers 515F and 907R were used to extract the sequences of V4 and V5 regions from SILVA database (69), followed by training to obtain a naive Bayes classifier.

Sequences that passed the quality control were denoised and merged using the DADA2 algorithm, using the parameters denoise-paired –i-demultiplexed-seqs –p-trunc-len-f 0 –p-trunc-len-r 0 –o-table –o-representative-sequences, prior to taxonomy classification with the classifier (70). DADA2 analysis resulted in ∼27,316 clean sequences in each sample (Data Set S1, tab 1).

Subsequently, the data were uploaded to MicrobiomeAnalyst without filtering to complete alpha diversity and beta diversity analysis (total sum scaling). Here, we present the results based on the analysis of the bacterial family level.

### Metagenomic sequencing.

The total DNA for 16S rRNA sequencing was also used for metagenomic sequencing on a MGISEQ-2000 platform (BGI, China), which generated paired-end 150-bp sequences. After the removal of adapters and sequences containing N bases, at least 10 Gb of high-quality clean sequences was obtained in each sample.

### Resistome profile.

Quality control of the raw sequences from metagenomics sequencing was performed with FastQC and Trimmomatic, which generated the clean sequences (71, 72). More specifically, the sequences were removed if they had 50% of consecutive low-quality bases (quality score ≤ 12), 10% ambiguous (N) bases, sequencing adapters, and duplication contaminations. After quality control, 10 to 20% of the raw sequences were removed, and additional sequencing was carried out to obtain at least 10 Gb of data for each sample. Then, the first 10 bp of those sequences were trimmed with Trimmomatic due to the instability of the sequencing platform during the initial sequencing process. DeepARG-SS was used in the calculation of relative abundance of ARGs. The copy number of ARG subtype genes, normalized by the copy number of 16S rRNA genes in the clean sequences, was calculated with the following thresholds: 80% identity of ARGs, 0.8 probability, an *E* value of 1e−10, 80% coverage, and 80% identity of 16S rRNA sequences (73). Discriminatory ARGs resulting in the unique occurrence pattern of resistome were identified by ExtraARG (74). More specifically, GSC was compared with NSC, whereas GST was compared against NST.

To identify the bacterial hosts carrying ARGs, taxonomic classification of ARGs was performed as described by Yin et al. (75), with minor modifications. The clean sequences were *de novo* assembled into contigs with default parameters using MEGAHIT (76). Afterwards, ORFs in the contigs were predicted via -p meta in Prodigal V2.6.3 (77). To characterize contigs carrying ARGs, the blastx mode of DIAMOND was used to annotate the ORFs with the database of DeepARG, with thresholds of an *E* value of 1e−10, 80% identity, and 70% coverage (78, 79). Contigs carrying ARG ORFs were obtained and sent for taxonomy classification by Kraken2 using the bacterial database (80). Details of contigs and ORFs are presented in Data Set S1, tab 8.

### Statistics.

Statistical tests and data visualization were accomplished by MicrobiomeAnalyst, GraphPad Prism 8 (GraphPad Software, Inc., USA), R 3.6.2, Origin 2020b (OriginLab Corporation, USA), and Past 4.03 (68, 81, 82). All the data were tested for normality using the D'Agostino-Pearson test (alpha = 0.05). To reveal significant differences in the Shannon index, the relative abundance of total ARGs, and the relative abundance of *Burkholderiaceae* with MDR genes among three or more soils (e.g., GSC versus DSC versus NSC, and GSC versus GST1, GST2, and GST3), a nonparametric Friedman test was performed (*P* < 0.05). Multiple comparisons were performed in pairwise analysis with the Benjamini-Hochberg method to control the false discovery rate (FDR; *q* < 0.1) (e.g., GSC versus DSC, GSC versus NSC, and DSC versus NSC).

To reveal significant difference in taxa and ARG types, the Friedman test was first performed (*P < *0.05) among three or more soils, e.g., GSC versus DSC versus NSC, followed by pairwise analysis using multiple Wilcoxon matched-pairs signed rank tests with the Benjamini-Hochberg method to control the FDR (*q *< 0.1), e.g., GSC versus DSC, GSC versus NSC, and DSC versus NSC. Furthermore, total sum scaling was performed prior to DESeq2 and LEfSe analysis. The trimmed mean of M-value transformation before edgeR analysis was carried out to identify differentially abundant taxa in MicrobiomeAnalyst. Analysis of similarities (ANOSIM) (*P < *0.05) and permutational analysis of variance (PERMANOVA) (*P < *0.05) were used in beta diversity analysis of taxa and ARG subtypes, respectively. Further, the sequential Bonferroni correction was used in pairwise analyses.

### Data availability.

The data sets supporting the results and discussion of this article were deposited in NCBI under BioProject accession no. PRJNA628860 for resistome analysis and accession no. PRJNA630011 for 16S rRNA analysis.
