# Supplementary figures and images for: Burkholderiaceae and Multidrug Resistance Genes Are Key Players in Resistome Development in a Germfree Soil Model
Source: mSystems. 2021 Nov 2;6(6):e00988-21. doi: 10.1128/mSystems.00988-21 (PMC8562478; doi:10.1128/mSystems.00988-21)

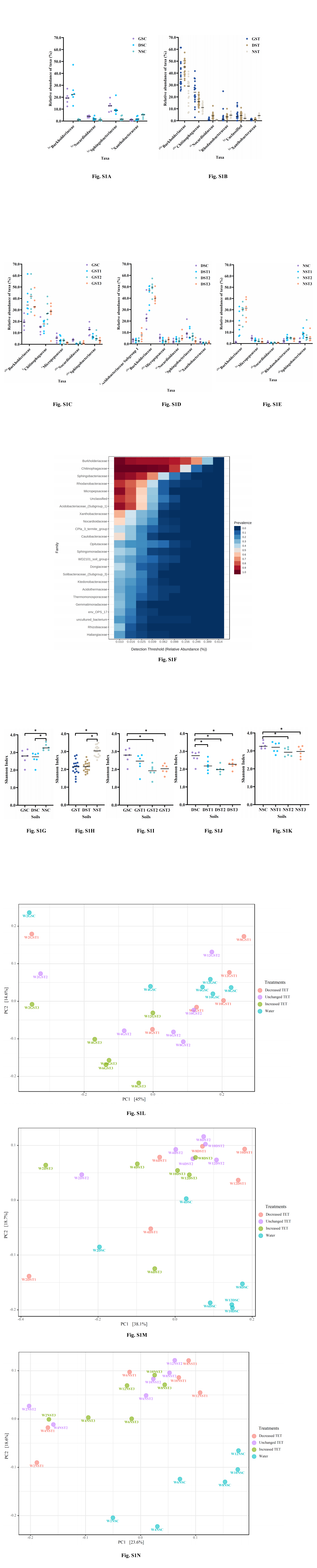

Supplement: FIG S1 [file msystems.00988-21-sf001.tif]

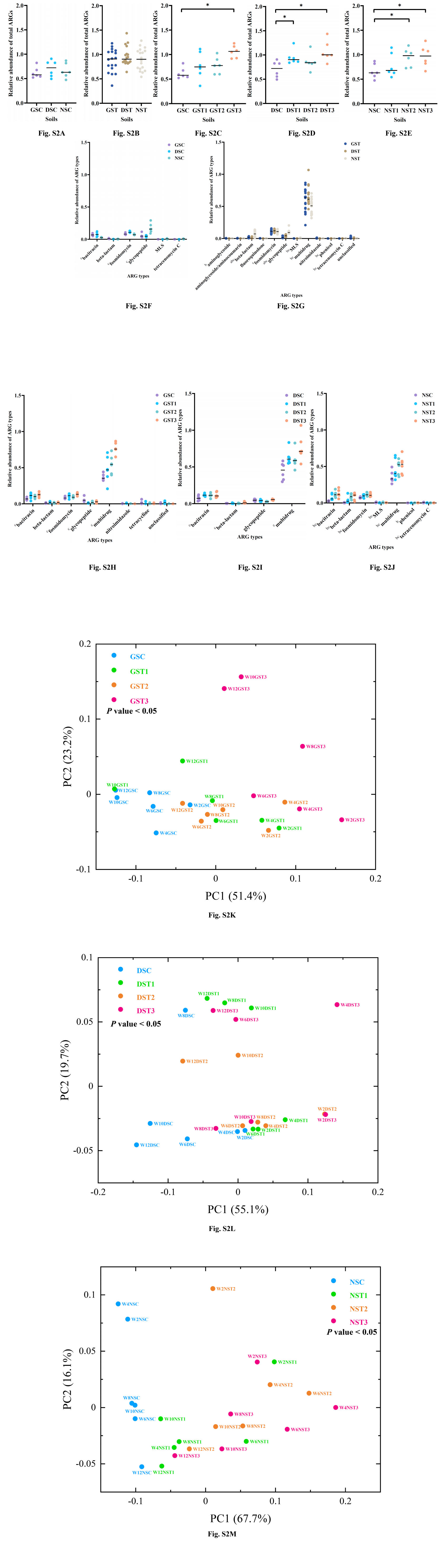

Supplement: FIG S2 [file msystems.00988-21-sf002.tif]

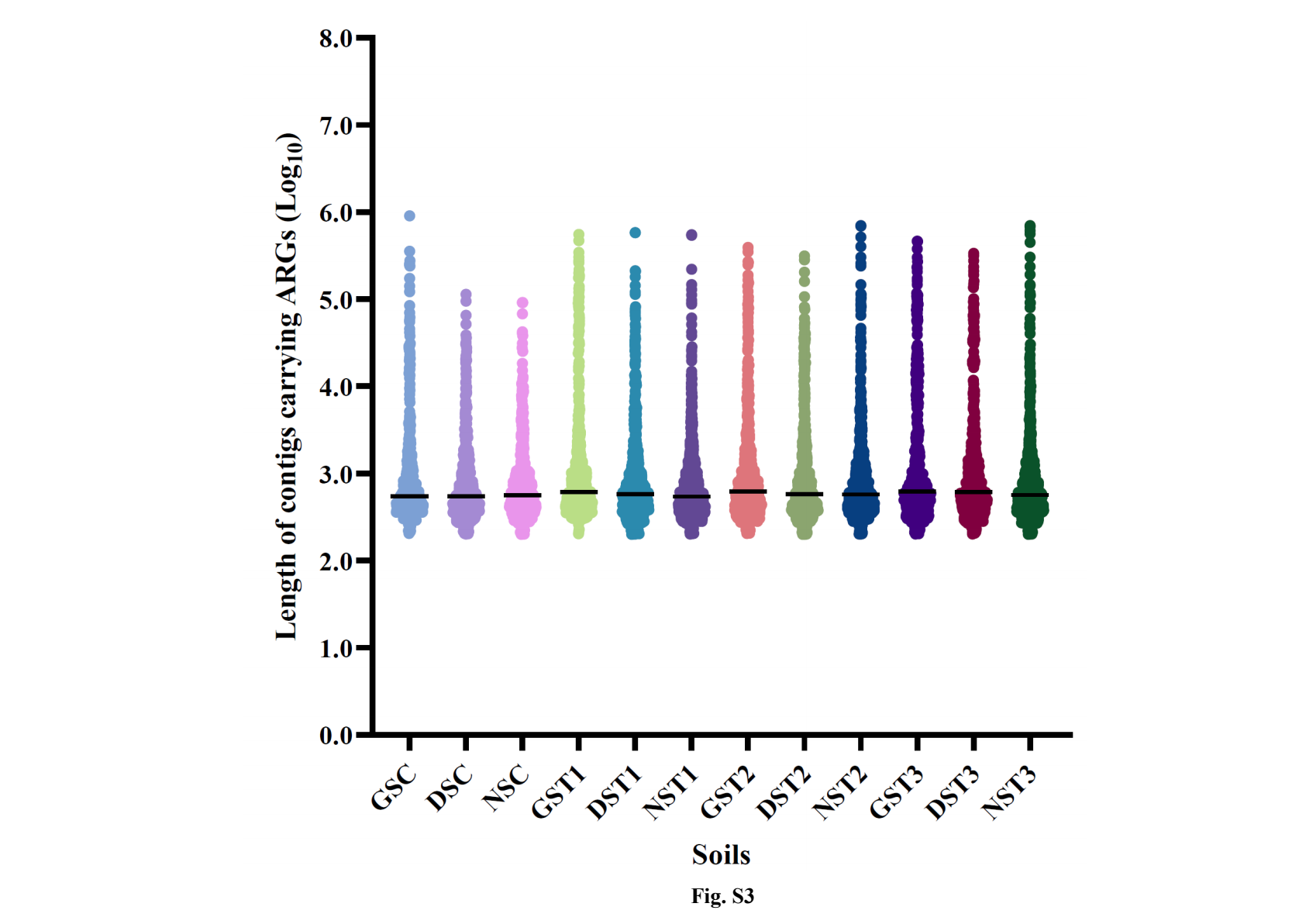

Supplement: FIG S3 [file msystems.00988-21-sf003.tif]
